# A case of ischemic stroke with hemorrhagic transformation associated with essential thrombocythemia and JAK-2 V617F mutation

**DOI:** 10.1186/s12883-022-02964-z

**Published:** 2022-11-17

**Authors:** Ran Yan, Donghua Mi, Xin Qiu, Zixiao Li

**Affiliations:** 1 Vascular Neurology, Department of Neurology, Beijing Tiantan Hospital, Capital Medical University, Fanyanglu Street, Beijing, 100070 China; 2 China National Clinical Research Center for Neurological Diseases, Beijing, China; 3 National Center for Healthcare Quality Management in Neurological Diseases, Beijing, China; 4 Center for Stroke, Beijing Institute for Brain Disorders, Beijing, China

**Keywords:** Ischemic stroke, Hemorrhagic transformation, JAK2 mutation, Essential thrombocythemia

## Abstract

**Background:**

Essential thrombocythemia (ET) is a rare cause of stroke. The V617F mutation in the Janus kinase 2 (JAK2) gene is one of the most typical mutations in ET and has been shown to be a risk factor for stroke, especially in younger people. However, to date, there have been few reports of intracranial thrombotic and hemorrhagic complications in patients with ET. Herein, we present a case of JAK2 gene mutation-associated ET in a patient who developed both ischemic and hemorrhagic stroke, and discuss potential underlying mechanisms.

**Case presentation:**

A 45-year-old Chinese male presented to our center with gradually developing weakness of the right limbs for 3 months. A computed tomography scan of the brain showed an area of infarction with hemorrhage in the left subcortical and corona radiata regions. High-resolution magnetic resonance imaging revealed a thrombosis on the surface of the atherosclerotic plaque. Digital subtraction angiography revealed an insect bite-like change in the C1 branch of the left internal carotid artery, which caused up to 50% stenosis. Blood tests showed continued elevation of the platelet and white blood cell counts. After consultation with a hematologist, a bone marrow biopsy was performed, which revealed proliferative bone marrow changes with numerous megakaryocytes and proliferative but mature granulocytes. Further genetic testing revealed a positive JAK2-V617F mutation. Therefore, the diagnosis of ET was confirmed according to the World Health Organization (WHO) 2016 diagnostic criteria. Finally, we decided to administer aspirin and hydroxyurea. The patient remained stroke free and the platelet levels were normal throughout the 1-year follow-up period.

**Conclusions:**

JAK2 mutations affect the proliferation and differentiation of blood cells through the JAK, signal transducer and activator of transcription pathway, which leads to changes in platelets and macrophages, and an increase in neutrophil extracellular traps, which may explain the patient’s ischemic and hemorrhagic changes. Further investigation of the underlying mechanisms may change the treatment strategy for such patients in the future.

## Background

Essential thrombocythemia (ET) is a rare clonal hematopoietic stem cell disorder and a classic myeloproliferative neoplasm. The Janus kinase 2 (JAK2) V617F mutation is often observed in patients with ET. JAK2 is a major cause of ischemic stroke and associated with thrombosis [1]. Ischemic strokes are frequent in patients with ET, whereas intracranial hemorrhagic complications are rare [2]. To date, there have been few reports of intracranial thrombotic and hemorrhagic complications in ET patients with the JAK2 V617F mutation. This article describes such a case. We also review the potential mechanisms that underly the patient’s ischemic and hemorrhagic changes, which may change the treatment strategy for such patients in the future.

## Case presentation

A 45-year-old man presented to our center with gradually developing weakness of the right limbs for 3 months. He underwent brain magnetic resonance imaging (MRI) at another hospital 3 months prior to admission, which showed an acute ischemic stroke of the left parietal lobe. Twenty days before admission, MRI showed cerebral and subarachnoid hemorrhages, although he had no new symptoms or exacerbation at that time. Ten days before admission, he presented with a sudden headache in the occipital region, difficulty in finding words, and unsteady walking. The patient did not complain of abdominal or bone pain.

At admission, his vital signs and general examination were normal. Mucocutaneous alterations were not observed. Neurologic examination revealed expressive aphasia and right-sided extremity weakness graded 4/5 on the Medical Research Council scale (total range, 0 [no movement is observed] to 5 [muscle contracts normally against full resistance]).

His medical history was unremarkable, with no history of vascular risk factors, including diabetes, hypertension, hyperlipidemia, cardiomyopathy, and atrial fibrillation. He also denied smoking, alcohol consumption, or illicit drug use. The patient’s father died of cerebral hemorrhage.

**Fig. 1:**
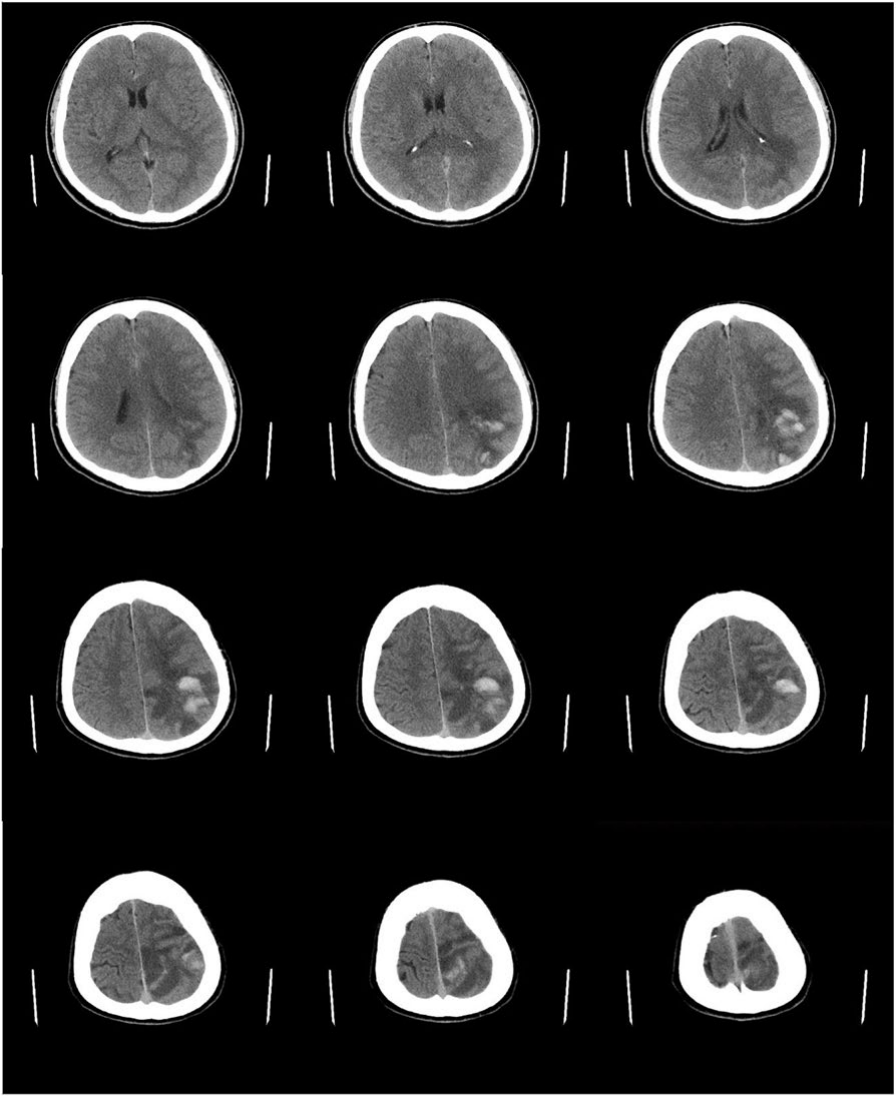
Computed tomography scan of the brain axial cuts shows a hypodense mixed with hyperintense lesion consist with cerebral infarction and hemorrhage transformation in the left subcortical and corona radiata region. The hemorrhage was more than 30% of the infarct area

**Fig. 2:**
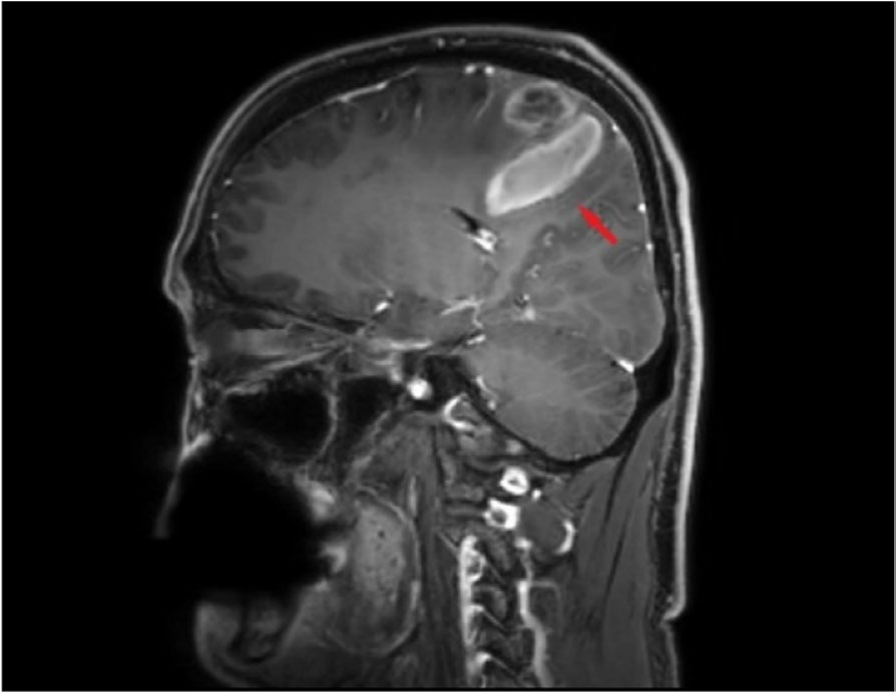
Enhanced magnetic resonance imaging shows meningeal and peripheral enhancement, but no significant enhancement in the hemorrhage area

**Fig. 3:**
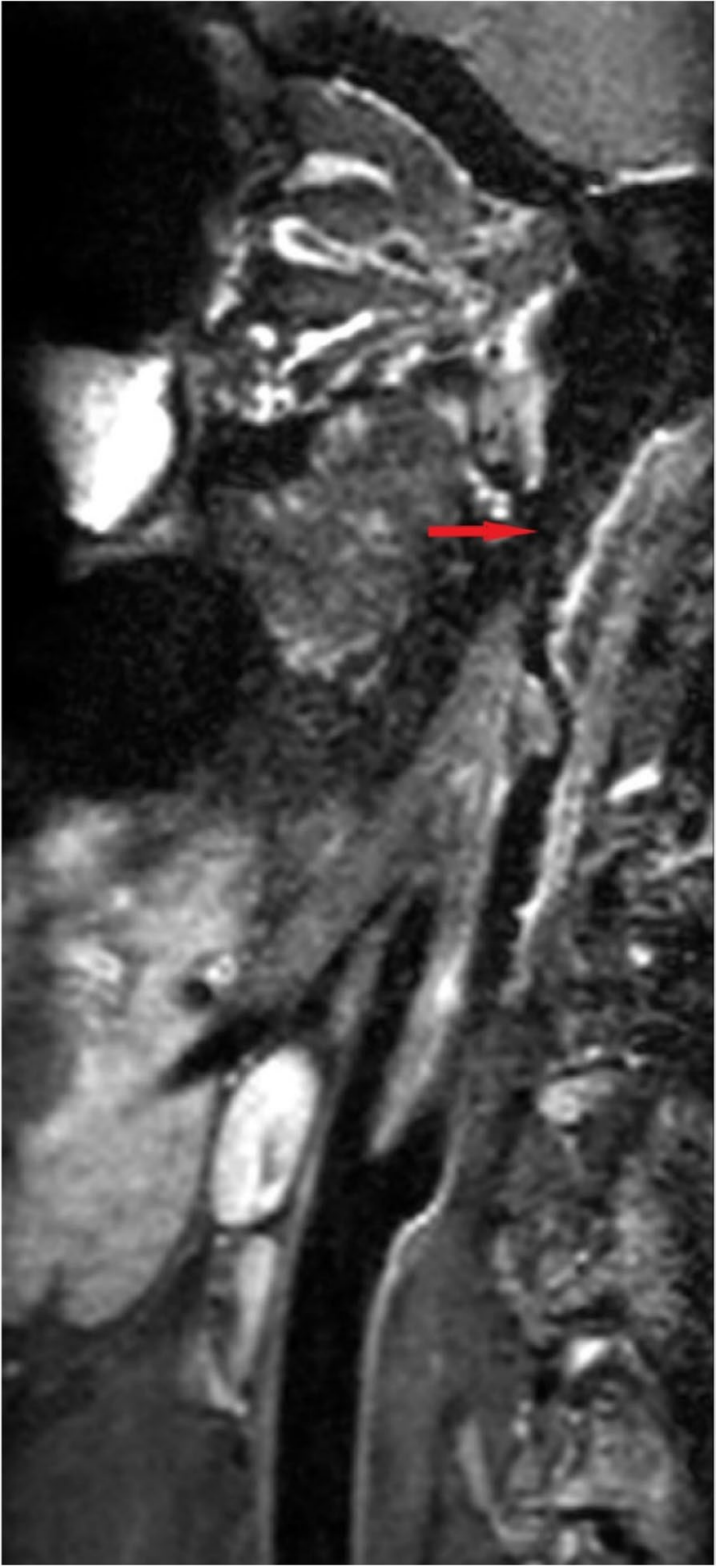
High-resolution magnetic resonance imaging shows enhanced signal on the surface of the atherosclerotic plaque, representing thrombosis, which has caused moderate to severe stenosis

**Fig. 4:**
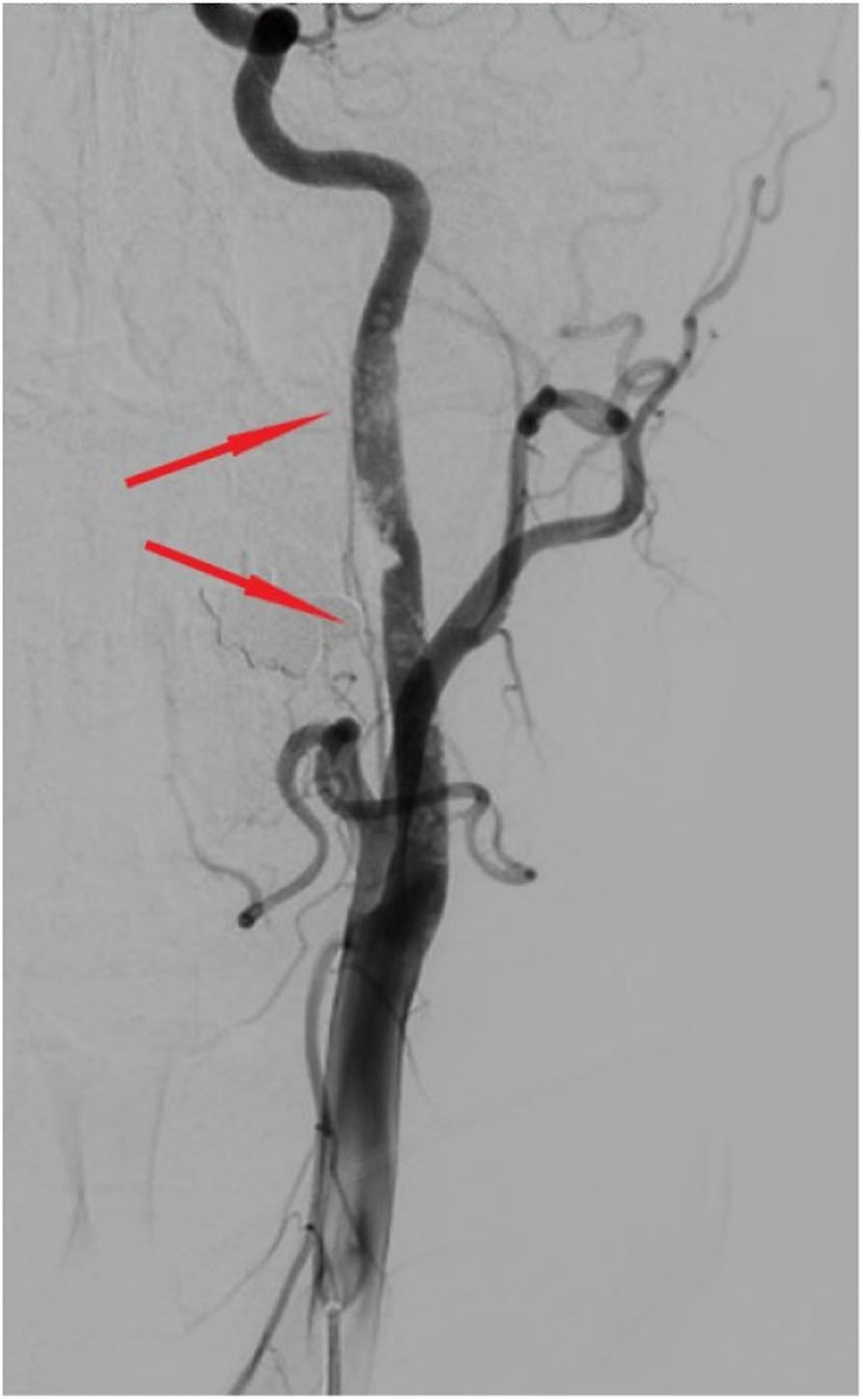
Digital subtraction angiography (DSA) shows a insect-bite-like changes of the whole segment of C1 branch of left internal carotid artery and caused stenosis up to about 50%

A computed tomography scan of the brain showed an area of infarction with hemorrhage in the left subcortical and corona radiata regions (Fig. 1), and the European Cooperative Acute Stroke Study classification was that of parenchymal hematomas 2 (PH2). MRI (Fig. 2) revealed meningeal and peripheral enhancement but no significant enhancement in the hemorrhage area. High-resolution MRI (Fig. 3) revealed a thrombosis on the surface of the atherosclerotic plaque. Digital subtraction angiography (DSA; Fig. 4) revealed an insect bite-like change in the C1 branch of the left internal carotid artery, which caused up to 50% stenosis. Cerebrovascular malformations and other carotid or intracranial arterial stenoses were excluded.

All blood test results were unremarkable, except for a continued elevation in the platelet (501 × 10^9^/L-601 × 10^9^/L) and white blood cell counts (Table 1), with normal coagulation function. Therefore, bone marrow biopsy and genetic testing were performed after consultation with a hematologist. Bone marrow biopsy revealed proliferative bone marrow changes, with numerous megakaryocytes and proliferative but mature granulocytes. Further genetic testing revealed a positive JAK2-V617F mutation.

**Table 1:** Platelets, white blood cells and hemoglobin during hospitalization

Date	Neurological symptoms	Platelets (× 10^9^/L)	White blood cells (× 10^9^/L)	Hemoglobin (g/L)
2019.07.01	expressive aphasia and right-sided extremity weakness graded 4/5 on the MRC scale	601	14.77	172
2019.07.03	Same as above	501	15.30	170
2019.07.09	Same as above	580	13.79	168
2019.07.15	Same as above	531	13.19	165

*MRC* the Medical Research Council

Myeloproliferative disease is a possible cause of complex cerebrovascular lesions. Therefore, the diagnosis of ET was confirmed according to the diagnostic criteria of the World Health Organization (WHO) 2016. After discussing with the hematologist, we decided to administer aspirin and hydroxyurea. After treatment, the patient remained stroke free (mRS score = 1/6, total range 0 [symptom-free] to 6 [dead]), and platelet levels were normal throughout the 1-year follow-up period.

## Discussion and conclusions

Our study provides a case report of ischemic stroke accompanied by hemorrhagic complications with a diagnosis of ET established from platelet counts, bone marrow biopsy, and genetic testing. The most frequent symptoms of ET include headache (27.5%) and abdominal or bone pain (5.5%); however, half of the patients with ET are asymptomatic [3], and 14% are diagnosed with arterial thrombosis. Patient management includes antiplatelet and cytoreductive therapy, as in the current case. Only a few published case reports of intracerebral hemorrhage in patients with ET and JAK2 V617F mutations exist [4–7].

Our patient had the JAK2 V617F mutation, which can be observed in 60% of patients with ET [8]. The JAK2 V617F mutation has been demonstrated to be associated with arterial and/or venous thrombosis [1, 9], and an increased risk of cardiovascular and cerebrovascular diseases including incident coronary heart disease [10] and stroke [11]. In our case, an insect bite-like change in the C1 branch of the left internal carotid artery was found using DSA, which may be a typical imaging manifestation of ET and JAK2 V617F, because the expression of JAK2 V617F promotes early lesion formation and increases complexity in advanced atherosclerosis, contributing to plaque instability [12].

The pathogenesis of hemorrhagic stroke in ET has not yet been fully elucidated. Previous studies have suggested that hemorrhagic complications of ET are associated with a highly elevated platelet count (> 1,000,000/mL) and are often associated with acquired Von Willebrand syndrome [5, 7]. However, in both cases, the platelet count was low. The JAK2 V617F mutation may cause stroke through thrombo-inflammatory mechanisms by affecting the proliferation of myeloid cells and leading to increased inflammatory responses, as JAK2 controls precursor cell maintenance and function in hematopoiesis [13]. The abnormal JAK2 V617F mutation activates the JAK, signal transducer and activator of transcription (STAT) conduction pathway and other signaling pathways, causing excessive phosphorylation of the downstream pathways; these are activated continuously without depending on exogenous cytokines, such as erythropoietin or platelet hormone, resulting in abnormal cell proliferation and differentiation.

Platelets, macrophages, and neutrophils are closely associated with the pathological process of JAK2 V617F-related stroke. Evidence has shown that the JAK2 V617F mutation affects quantitative and/or qualitative platelet traits [14]. The fraction of immature platelets was higher in carriers of the JAK2 V617F mutation than in non-carriers, and immature platelets were more active than mature platelets. In mice that express JAK2 V617F selectively in macrophages, increased proliferation and glycolytic metabolism in JAK2 V617F macrophages leads to DNA replication stress and activation of the AIM2 inflammasome, thereby aggravating atherosclerosis [15]. It has also been shown that neutrophils play a pro-atherosclerotic role in early atherosclerotic formation [12]. Neutrophils accumulate in the peri-infarct cortex at all stages of ischemic stroke. JAK2 V617F mutations increase the expression of peptidylarginine deiminase 4 (PAD4) on neutrophils in vivo. PAD4 is an enzyme essential for neutrophil extracellular trap (NET) formation that may lead to thrombosis [16].

Hemorrhagic stroke is rare in JAK2-associated cerebral thrombosis [8]. However, in our case, it occurred even though the platelet count continued to increase. Blood–brain barrier disruption plays an important role in the development of neurological dysfunction in ischemic stroke and is closely associated with subsequent hemorrhagic transformation [17, 18]. A recent study also showed increased NET formation in venous thrombi in JAK2 V617F mice [19]. One possible explanation is that PAD4 is upregulated in peri-ischemic brains and its overexpression induces an increase in NET formation, which is accompanied by increased blood–brain barrier damage [20].

The identification of the pathophysiological mechanisms remains challenging. Further insights into the functional consequences of JAK2 mutations may enable personalized risk assessment, not only with regard to malignancies, but also in relation to thrombotic vascular disease. Our case suggests that the diagnosis of ET should be considered when identifying cerebrovascular disease accompanied by platelet increase in clinical practice. Clinicians should also be alerted when the imaging examination shows insect bite-like changes. Antiplatelet therapy combined with hydroxyurea is effective in such patients.

Our study had several limitations. First, the hemorrhagic lesion with edema, the slow course, and subarachnoid bleeding are likely due to a venous occlusion rather than an arterial plaque, but imaging of the venous system, such as magnetic resonance venography or computed tomography venography, was not available. Second, platelet counts before stroke and plasma Von Willebrand Factor (VWF) levels were not monitored. These limitations restrict further discussion of the cause of the disease.

## Conclusion

We present a case of JAK2 gene mutation-associated ET in a patient who developed both ischemic and hemorrhagic stroke. JAK2 mutations affect the proliferation and differentiation of blood cells through the JAK/STAT pathway, which leads to changes in platelets and macrophages and an increase in NETs, which may explain the patient’s ischemic and hemorrhagic changes. Further exploration of the underlying mechanisms may change the treatment strategy for such patients in the future; for example, the JAK2 inhibitor, ruxolitinib, can effectively inhibit NET formation and reduce thrombosis in mice. Such a treatment may be used clinically in the future for certain patient populations.

## Data Availability

Not applicable.

## Abbreviations

CT: Computed tomography
DSA: Digital subtraction angiography
ET: Essential thrombocythemia
JAK2: Janus kinase 2
MRI: Magnetic resonance imaging
NET: Neutrophil extracellular trap
PAD4: Peptidylarginine deiminase 4
VWF: Von Willebrand Factor
